# Supplementary material for: Comparative genomics provides new insights into the diversity, physiology, and sexuality of the only industrially exploited tremellomycete: Phaffia rhodozyma
Source: BMC Genomics. 2016 Nov 9;17:901. doi: 10.1186/s12864-016-3244-7 (PMC5103461; doi:10.1186/s12864-016-3244-7)
Supplement: Additional file 6: — List of orphan genes with links to PFAM (related to Additional file 1: Table S1). (ZIP 1428 kb) [file 12864_2016_3244_MOESM6_ESM.zip › PFAM_UNIQUE/G04975_P.html]

#                                                                            --- full sequence --- -------------- this domain -------------   hmm coord   ali coord   env coord
  
# target name        accession   tlen query name           accession   qlen   E-value  score  bias   #  of  c-Evalue  i-Evalue  score  bias  from    to  from    to  from    to  acc description of target
  
#------------------- ---------- ----- -------------------- ---------- ----- --------- ------ ----- --- --- --------- --------- ------ ----- ----- ----- ----- ----- ----- ----- ---- ---------------------
  
G04975\_P             -            446 ECM11                PF15463.1    139    0.0024   16.8   2.2   1   1   2.9e-06    0.0085   15.0   2.1    67   124   370   427   340   430 0.87 - Extracellular mutant protein 11  
G04975\_P             -            446 GAS                  PF13851.1    201     0.044   11.6   1.2   1   1   0.00037     0.071   11.0   1.2    79   150   358   429   354   433 0.94 - Growth-arrest specific micro-tubule binding
